# Chronic Pain Opioid-Maintained Patients Receive Less Analgesic Opioid Prescriptions

**DOI:** 10.3389/fpsyt.2018.00335

**Published:** 2018-07-23

**Authors:** Jessica Delorme, Chouki Chenaf, Celian Bertin, Marie Riquelme, Alain Eschalier, Denis Ardid, Nicolas Authier

**Affiliations:** ^1^Université Clermont Auvergne, CHU Clermont-Ferrand, Inserm, Neuro-Dol, Service de Pharmacologie médicale, Centres Addictovigilance et Pharmacovigilance, Centre Evaluation et Traitement de la Douleur, Clermont-Ferrand, France; ^2^Observatoire Français des Médicaments Antalgiques/French Monitoring Centre for Analgesic Drugs, Université Clermont Auvergne – CHU Clermont-Ferrand, Clermont–Ferrand, France; ^3^Faculté de Médecine, Institut Analgesia, Clermont-Ferrand, France

**Keywords:** opioid analgesics, chronic non-cancer pain, opioid-maintained patients, methadone, buprenorphine

## Abstract

Treating pain and opioid use disorder represents a clinical challenge. While most studies that have assessed opioid analgesic use in opioid substitution treatment (OST) patients primarily address opioid analgesic misuse (1, 2), only few studies focused on OST patients assessed the prescription of analgesic opioids for chronic pain. We sought to compare the prevalence of analgesic opioid prescription (AOP) in two groups of chronic non-cancer pain (CNCP) patients: OST patients vs. the general population. This was a population-based cross-sectional study based on the French national healthcare claims database SNIIRAM (Système National d'Informations Inter-Régimes de l'Assurance Maladie) covering over 66 million people (98.8% of the French population). Overall, 67,173 participants ≥15 years old undergoing continuous OST in 2015 (“OST patients” group) were included and age- and gender-matched by means of a 1:1 ratio with 67,173 patients without OST (“control” group). In each group, patients with cancer conditions were excluded and those having received opioid and non-opioid analgesics for at least 3 months were identified (CNCP patients). Compared to control patients, CNCP OST patients received less AOP (47.8 vs. 68.0%, *p* < 0.0001) and more often non-opioid prescription (52.2 vs. 32.0%, *p* < 0.0001). In multivariate analysis, CNCP OST patients were 2.7 times less likely to be prescribed analgesic opioids (adjusted odds ratio [OR] = 2.7 [2.42–3.01], *p* < 0.0001) than control patients. AOP correlated in CNCP OST patients with: age ≤ 40 years old, female gender, low-income status, methadone-maintained treatment, mental health disorder, hepatitis C virus (HCV) infection, and alcohol abuse disorder. Opioid analgesics were less often prescribed in CNCP OST patients. AOP prevalence was 2.7-fold lower than in the general population. Chronic pain management in OST patients needs to be reinforced through additional physician training and a multidisciplinary approach.

## Introduction

The use of analgesic opioids has dramatically increased over the last decade, particularly in North America, Europe, and Oceania (3–6). This increased prescription of analgesic opioids is partly related to the promotion of opioid use in chronic non-cancer pain (CNCP) management, despite the remaining controversy due to high-level scientific evidence demonstrating their weak long-term benefit (6–8). Worldwide, from 19 to 32% of general population suffered from chronic pain (9–15). Focusing on patients receiving opioid substitution treatment (OST), studies have revealed higher prevalence of chronic pain, reported in 29–68% of methadone patients (16–24) and 40–49% of buprenorphine patients (19, 23, 25).

Treating both pain and addiction proves to be a significant challenge, and caregivers commonly report that all their diagnostic and therapeutic decisions are subject to ambiguity (26). While most studies examining opioid analgesic use in OST patients have addressed opioid analgesic misuse (1, 2), only few have specifically focused on OST patients who are prescribed analgesic opioids for chronic pain; the prevalence in this opioid-dependent population of analgesic opioid prescription (AOP) ranged from 43 to 47% (17, 21, 27).

Pain management in OST patients is often stressful for primary care physicians (28), and they may be wary of prescribing opioid analgesics due to the potential risks of overdose (3, 29), diversion, and abuse (21). Inadequate AOP may both undertreat the pain and precipitate withdrawal symptoms (30). A recent study suggested that pain in methadone-maintained patients was undertreated: 54% of patients received no analgesic treatment, 24% analgesic opioids, and 22% non-opioid analgesics (31–33).

Furthermore, according to Whitehead et al. opioid prescriptions in patients with concomitant pain and substance abuse disorders were less frequent than in patients with pain only (34). As a result, OST patients are often unsatisfied with the insufficient healthcare they are receiving, which subsequently leads them to use licit or illicit opioids to relive their pain properly (17, 25, 27, 35).

This study sought to estimate and compare the prevalence of analgesic (opioid and non-opioid) prescription between CNCP OST patients and the non-opioid-dependent population with general CNCP, as well as to identify factors associated with AOP.

## Methods

### Design

This was a population-based cross-sectional study using the French national claims database, SNIIRAM (*Système National d'Informations Inter-Régimes de l'Assurance Maladie*), covering almost 99% of the French population (36). This database is completely anonymous and each patient has a unique anonymized identification number.

### Data

The SNIIRAM database contains: (1) demographic data: gender, date of birth, information on the complementary private insurance coverage indicating low-income status; (2) presence of chronic disease, represented by a list of 30 long-term diseases (*Affection Longue Durée* [ALD]) with their associated ICD-10 (International Classification of Disease, version 10) codes; 3) medications, recorded as dispensed preparation packs including ATC (Anatomical Therapeutic Classification) codes, with pack descriptions including number of tablets, number of packs, dates of prescription and dispensation, as well as prescriber's specialty; (4) date and duration of hospital admissions, including both primary, related, and secondary diagnoses, with ICD-10 codes.

We were authorized to use the SNIIRAM database by the French national commission on information technologies and liberties (the French national data protection agency CNIL). As the SNIIRAM database is fully anonymized, no ethic committee approval is necessary.

### Participants

We selected all opioid-maintained patients, male and female, aged ≥15 years old, having received at least one OST prescription (methadone [MTD] or high-dose buprenorphine [HDB] ± naloxone) between January 1st and December 31st 2015. Among these patients, those receiving continuous HDB or MTD treatment throughout 2015 were included, composing the “OST patients” group. Continuous treatment was defined as an interval between two consecutive dispensations ≤35 days for HDB and MTD capsules and ≤18 days for MTD syrup.

This 35-day threshold corresponded to the sum of the 28-day maximum prescription duration for MTD capsule or HDB plus 7 days more to allow more precision for the identification of prescription interruption (+25% of 28 days) (37, 38). Similarly, the 18-day threshold corresponded to the sum of the 14-day maximum prescription duration for MTD syrup plus 4 days more (+25% of 14 days). The “OST patients” group was age- and gender-matched on a 1:1 ratio with a control group constituted of male and female patients aged ≥15 years old who had received no OST dispensation in 2015 (“General Population” control group). In each group, patients with cancer conditions were excluded and those treated continuously for at least 3 months with analgesics (interval between two consecutive opioid or non-opioid analgesic dispensations ≤ 45 days) were defined as CNCP patients.

### Measures

Several socio-demographic information were retrieved from this database: year of birth, gender, low-income status, and date of death. OST patients were selected through specific ATC codes: N07BC01, NO7BC51 for HDB and N07BC02 for methadone. Several comorbidities were recorded: human immunodeficiency virus (HIV) infection (ICD-10 code “B24”), hepatitis C virus (HCV) infection (ICD-10 codes “B182” and “B171”), and hepatitis B virus (HBV) infection (ICD-10 codes “B180,” “B181,” and “B16”). Alcohol use disorder was identified either by a specific ICD-10 code (“F10”) or at least one dispensation of acamprosate, disulfiram, or naltrexone (N07BB ATC code). Concomitant prescriptions of opioid analgesics were identified through specific ATC codes N02AA01 and N02AA51 (morphine), N02AB03 (fentanyl), N02AA05 and N02AA55 (oxycodone), N02AX02, and N02AX52 (tramadol), N02AA59 and N02AA79 (codeine), N02AA08, and N02AA58 (dihydrocodeine), N02AA02 (opium). Concomitant prescription of non-opioid analgesics included nefopam (ATC code N02BG06), paracetamol (N02BE01), non-steroidal anti-inflammatory drugs (M01A), and triptans (N02CC). ATC codes for psychotropic drugs were also recorded: N06A (antidepressants), N05A, except N05AN (antipsychotics), N05AN, N03AG01, and N03AG02 (mood stabilizers), N05B (anxiolytics), and N05C (hypnotics). The total quantity of analgesic opioids that were consumed was also calculated, each opioid converted to an oral morphine equivalent (OME) dose (39).

### Analyses

Patients' characteristics were presented as mean ± standard deviation [min-max] or as median [interquartile range] for continuous data and as the number of patients and associated percentages for categorical parameters. Comparisons of patient characteristics between the “CNCP OST patients” group and “the CNCP control” group were performed using the chi-squared test or the Fisher's test procedure test for categorical variables and *t*-test for quantitative variables, when appropriate.

To determine the influence of various factors associated with AOP in CNCP patients, a univariate logistic regression model was performed. The associated *p*-values were computed with their corresponding odds ratios (ORs) and their 95% confidence intervals (95% CI).

To study the risk factors associated with AOP, a multivariate logistic regression analysis was performed. All clinically-relevant variables or other variables associated with *p* < 0.25 in univariate analysis were included in the model. The corresponding adjusted ORs were calculated with their 95% CIs. A second univariate and multivariate analysis was then conducted to determine the factors associated with AOP in the subgroup of CNCP OST patients. SAS software for Windows Version 9.3 (SAS Institute, North Carolina, USA) was used for all statistical analyses.

## Results

**Figure 1:**
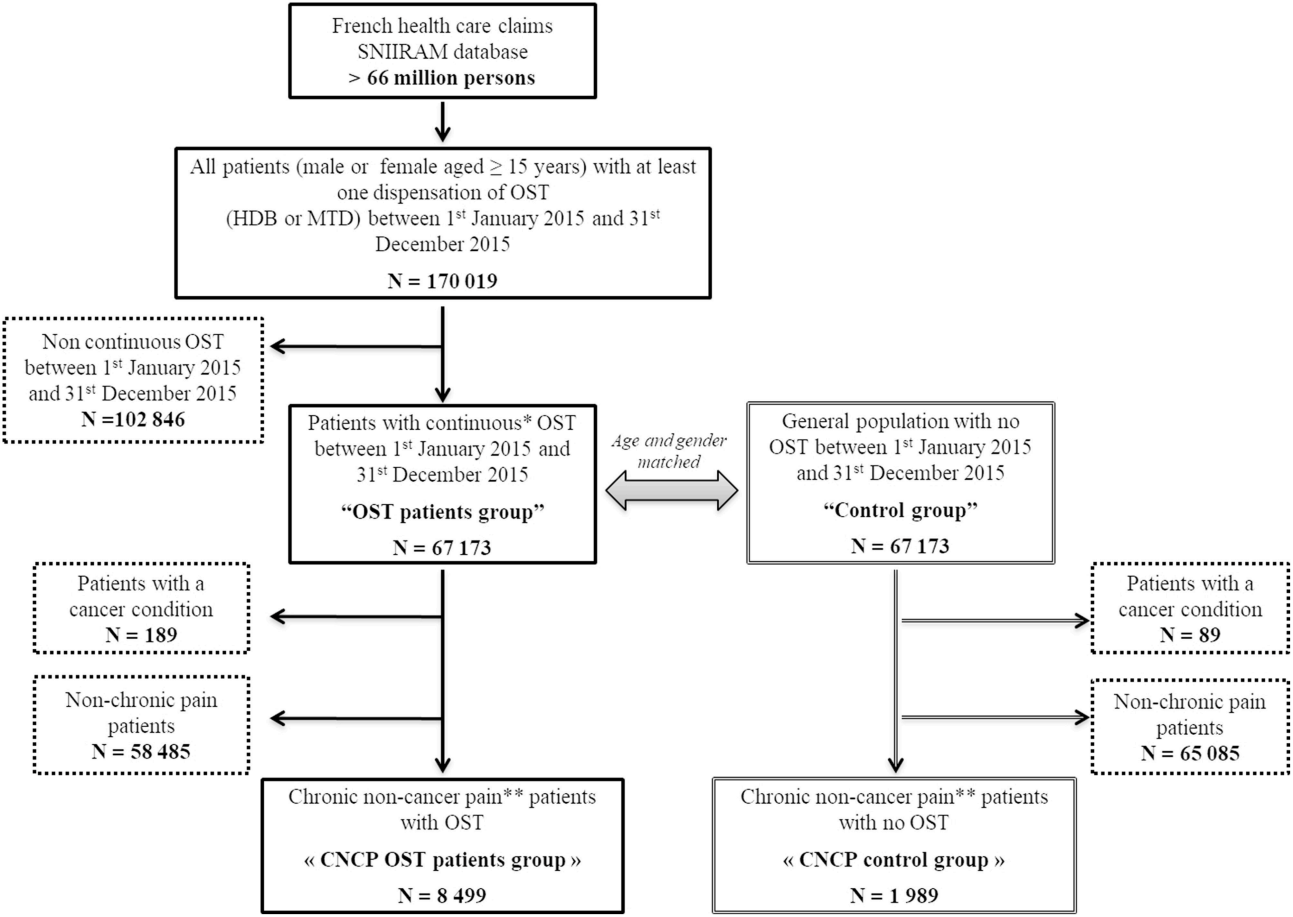
Flow chart. OST, opioid substitution treatment; HDB, buprenorphine; MTD, methadone; CNCP, chronic non-cancer pain; SNIIRAM, Systeme National d'Infonnations Inter-Regimes de I'Assurance Maladie. ^*^Interval between two consecutive dispensations ≤35 days for HDB and MTD capsule and ≤l8 days for MTD syrup. ^**^Patients treated continuously for at least 3 months with opioid analgesics and non-opioid analgesics (interval between two consecutive analgesic dispensations ≤45 days).

We identified 67,173 patients continuously treated with OST (OST patients group) (67% HDB and 33% MTD) in 2015, age- and gender-matching them with 67,173 patients from the general population without OST or any diagnosed opioid abuse disorder (control group) (Figure 1). After matching, the mean age was 40.3 ± 8.6 years [18-88] and the majority of patients were male (77.7%). Overall, 8,499 and 1,989 CNCP patients were identified from the OST patients group and control group, respectively (12.6 vs. 3.0%, *p* < 0.0001).

### Patient characteristics

Compared to the CNCP control group (Table 1), CNCP OST patients were younger (42.9 ± 8.6 yrs vs. 44.9 ± 8.8 yrs, *p* < 0,0001), more frequently male (71.3 vs. 65.1%, *p* < 0.0001), more frequently HIV-positive (2.4 vs. 1.0%, *p* < 0.001) or HCV infected (7.5 vs. 0.5%, *p* < 0.0001), and more often exhibited alcohol use disorders (7.9 vs. 4.0%, *p* < 0.0001) and mental health disorders (80.7 vs. 57.5%, *p* < 0.0001). The prevalence of low-income status and HBV infection were not statistically different between the two groups.

**Table 1:** Comparison of characteristics between CNCP OST patients group and CNCP control group in 2015.

	**CNCP OST patients group**	**CNCP control group**	***P*-value**
	***N = 8,499***	***N = 1,989***	
	**% (*N*)**	**% (*N*)**	
Age (years), mean (±SD)	42.9 ± 8.6	44.9 ± 8.8	< 0.0001
**AGE CLASS**			
≤40	37.9 (3,218)	30.6 (608)	< 0.0001
> 40	62.1 (5281)	69.4 (1381)	
Male gender	71.3 (6,059)	65.1 (1,295)	< 0.0001
Low-income status	23.7 (2,017)	25.7 (511)	0.07
HIV infection	2.4 (200)	1.0 (20)	0.0002
HCV infection	7.5 (634)	0.5 (10)	< 0.0001
HBV infection	0.3 (22)	0.4 (8)	0.28
Alcohol use disorder	7.9 (670)	4.0 (79)	< 0.0001
Mental health disorder	80.7 (6,857)	57.5 (1,143)	< 0.0001
Number of different OST prescribers, median [IQR]^a^	2 [1-2]	–	–
Buprenorphine	68.9 (5,857)	–	–
*Buprenorphine ± naloxone*	4.1 (346)	–	–
Methadone	30.9 (2,617)	–	–
*Capsule*	66.5 (1,510)	–	–
*Syrup*	33.5 (761)	–	–
Switch buprenorphine/methadone	0.2 (25)	–	–

*CNCP, chronic non-cancer pain; OST, opioid substitution treatment; HIV, human immunodeficiency virus; HCV, hepatitis C virus; HBV, hepatitis B virus; IQR, interquartile range (25th and 75th percentiles)*.

a *Total number of different OST prescribers used by opioid-maintained patients during 2015*.

The mean buprenorphine dose administered was significantly higher in CNCP OST patients than in non-CNCP OST patients (11.3 ± 11.5 mg [0.1–296.6] vs. 9.2 ± 9.3 mg [0.1–237.3], *p* < 0.0001), as was the mean methadone dose administered (syrup: CNCP OST: 59.8 ± 36.9 mg vs. non-CNCP OST: 52.7 ± 30.5 mg, *p* < 0.0001; capsule: CNCP OST: 61.2 ± 49.1 mg vs. non-CNCP OST: 53.6 ± 45.2 mg, *p* < 0.0001).

### Opioid and non-opioid analgesic prescription

Compared to CNCP control patients, CNCP OST patients were significantly less likely to be prescribed opioid analgesics (47.8 vs. 68.0%, *p* < 0.0001), both in terms of strong opioids (4.6 vs. 8.5%, *p* < 0.0001, with less frequent prescription of fentanyl, morphine, and oxycodone) and weak opioid prescriptions (46.1 vs. 65.4%, *p* < 0.0001, with less frequent prescription of codeine, tramadol, and opium) (Table 2). The median OME cumulative dose was significantly greater in CNCP control patients than in CNCP OST patients (1800 mg [450–6768] vs. 968 mg [300–4350], respectively, *p* < 0.0001).

**Table 2:** Comparison of analgesic drug prescription between chronic non-cancer pain opioid substitution treatment (CNCP OST) patients group and CNCP control group.

	**CNCP OST patients group**	**CNCP control group**	***P*-value**
	***N = 8,499***	***N = 1,989***	
	**% (*N*)**	**% (*N*)**	
**Opioid analgesic use**	47.8 (4,065)	68.0 (1,352)	< 0.0001
***Strong opioids***	4.6 (394)	8.5 (169)	< 0.0001
*Fentanyl*	0.6 (49)	1.4 (28)	< 0.0001
*Morphine*	3.2 (273)	5.2 (103)	< 0.0001
*Oxycodone*	1.3 (106)	3.4 (67)	< 0.0001
***Weak opioids***	46.1 (3,921)	65.4 (1,300)	< 0.0001
*Codeine*	19.0 (1,618)	32.1 (638)	< 0.0001
*Tramadol*	28.3 (2,403)	39.7 (790)	< 0.0001
*Opium*	13.0 (1107)	21.4 (425)	< 0.0001
**Exclusive non-opioid analgesic use**	52.2 (4,434)	32.0 (637)	< 0.0001
***NSAIDs***	35.8 (3,044)	20.7 (412)	< 0.0001
***Paracetamol***	48.0 (4,073)	29.1 (579)	< 0.0001
***Nefopam***	1.6 (132)	0.4 (8)	< 0.0001
***Triptans***	8.1 (687)	10.0 (200)	0.004

*CNCP, chronic non-cancer pain; OST, opioid substitution treatment; NSAIDs, non-steroidal anti-inflammatory drugs*.

Conversely, CNCP OST patients more often received non-opioid analgesics compared to CNCP control patients (52.2 vs. 32.0%, *p* < 0.0001), including more frequent prescriptions of NSAIDs, paracetamol, and nefopam. Triptans were less frequently employed in CNCP OST patients.

Different prescribers for OST and opioid analgesics were found in 81.7% (82.9% for weak opioids and 98% for strong opioids) and 47.1% for OST and non-opioid analgesic prescription. Compared to CNCP control patients, CNCP OST patients received a lower median number of different opioid prescriptions [4 (1–11) vs. 6 (2–12), *p* < 0.0001].

### Factors associated with opioid analgesic prescription

In univariate analysis, the factors associated with AOP were female gender (OR: 1.17 [95% CI: 1.07–1.27]), alcohol use disorder (OR: 1.44 [1.24–1.67]), mental health disorder (OR: 1.32 [1.20–1.44]), and OST (OR: 2.32 [2.09–2.57]). Other covariates that were found to be significant at the *p* < 0.25 level were: low-income status (OR: 0.93 [0.85–1.02]), HCV infection (OR: 1.12 [0.95–1.31]), HIV infection (OR: 0.84 [95% CI: 0.64–1.09]), and age ≤ 40 years (OR: 1.05 [0.97–1.14]). Conversely, HBV infection (OR: 1.22 [95% CI: 0.59–2.52], *p* = 0.59), was not significantly associated with AOP.

**Figure 2:**
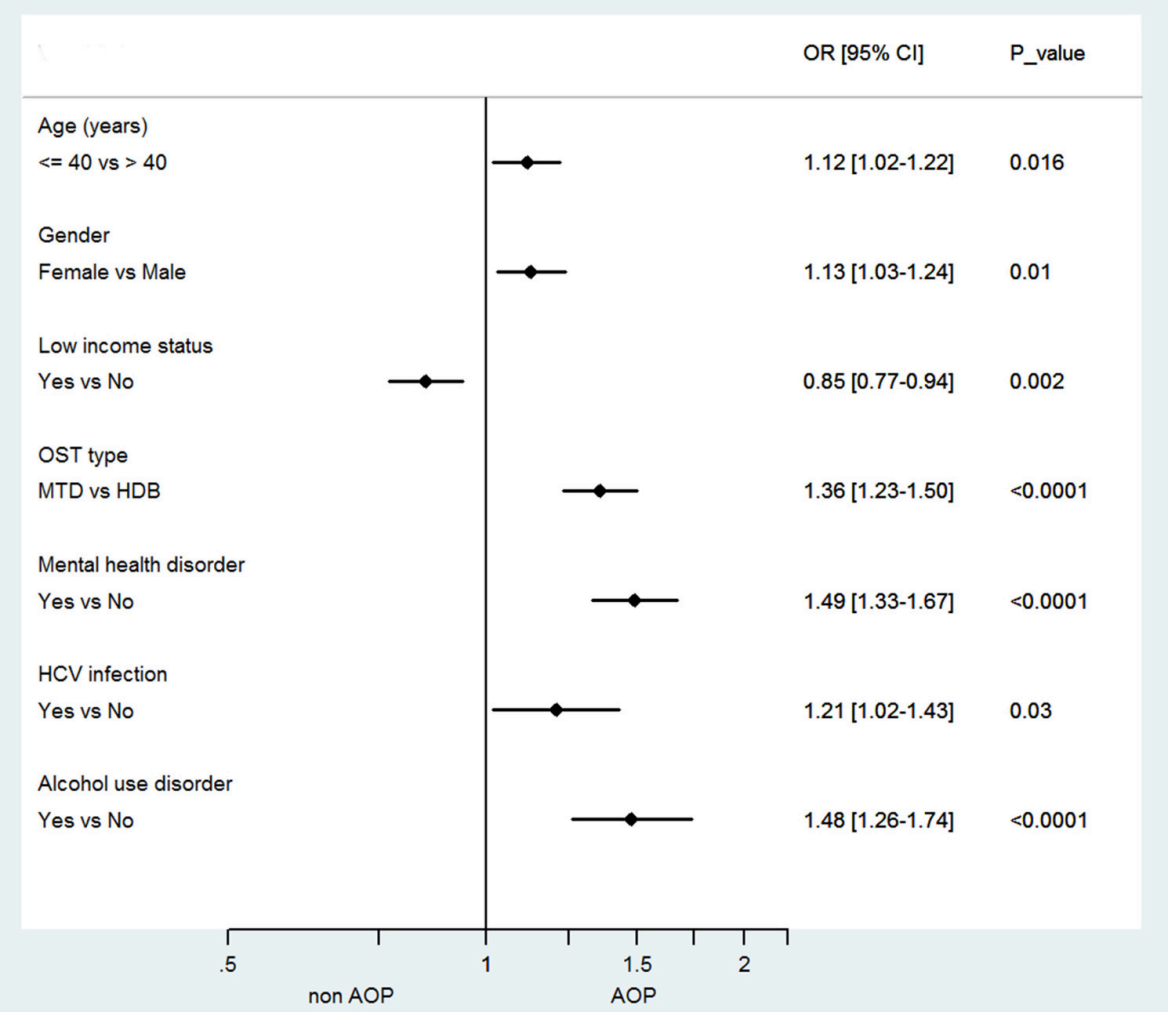
Forest plot of factors associated with analgesic opioid prescription in chronic non-cancer pain opioid substitution treatment (CNCP OST) patients. OR, odds ratio; CI, confidence interval; AOP, analgesic opioid prescription; MTD, methadone; HDB, buprenorphine, HCV, hepatitis C virus; OST, Opioid Substitution Treatment.

On multivariate analysis (Figure 2), there was still significant correlation with age ≤ 40 years (OR: 1.13 [1.04–1.23]), female gender (OR: 1.11 [1.02–1.20]), low-income status (OR: 0.87 [0.79–0.95]), mental health disorder (OR: 1.60 [1.46–1.76]), HCV infection (OR: 1.29 [1.09–1.52]), and alcohol use disorder (OR: 1.42 [1.22–1.65]). Compared to CNCP control patients, CNCP OST patients were 2.7 times less likely to be prescribed analgesic opioids (adjusted OR = 2.7 [2.42–3.01]).

### Factors associated with opioid analgesic prescription in the subgroup of CNCP OST patients

In multivariate analysis, age ≤ 40 years (OR: 1.12 [1.02–1.22]), female gender (OR: 1.13 [1.03–1.24]), low-income status (OR: 0.85 [0.77–0.94]), methadone treatment (OR: 1.36 [1.23–1.50]), mental health disorder (OR: 1.49 [1.33–1.67]), HCV infection (OR: 1.21[1.02–1.43]), and alcohol use disorder (OR: 1.48 [1.26–1.74]), were significantly correlated with AOP in chronic pain opioid-maintained patients (Figure 2).

## Discussion

To the best of our knowledge, the present nationwide study is the first that specifically compared opioid and non-opioid analgesic prescription in CNCP patients with or without OST. The prevalence of AOP was lower in the CNCP OST patients compared to CNCP control patients (47.8 vs. 68.0%). We observed similar prevalence of AOP in CNCP OST patients as that reported in prior studies, where prevalence ranged between 43 and 47% (17, 21, 27). Furthermore, following adjustment for age, gender, low-income status, mental health disorder, HCV infection, and alcohol use disorder, our findings revealed a 2.7-fold decrease in the prevalence of AOP in CNCP OST patients compared to CNCP control patients. Conversely, our study demonstrated analgesic non-opioids were more frequently administered in CNCP OST patients (52.2%) compared to CNCP control patients (32.0%). These findings were also consistent with prior studies, where this prevalence ranged from 48.9 to 64.1% in opioid-maintained patients (17, 25, 27).

Overall, chronic pain opioid-maintained patients received less AOPs and a number of hypotheses may be assumed.

### Opiophobia

The first hypothesis may be linked to the reluctance to prescribe analgesic opioids which is known as “opiophobia,” and many caregivers refuse to prescribe opiates at all, or limit prescriptions so far as to never provide enough to effectively relieve pain, and this phenomena is widespread across the world (40–43). Recognizing the potential risk from opioids, an international committee of pain specialists including physicians and healthcare policy specialists concluded that “*irrational fear of the drugs often impedes their appropriate use*” (44). Many physicians have admitted they were uncomfortable with the safe prescription of opioids (28) and have questioned their own practice of analgesic opioid prescribing. There are several reasons why physicians might not prescribe analgesic opioids in OST patients.

First, the use of analgesic opioids in addition to an OST may result in opioid abuse disorder relapse. A recent study showed that the risk of reporting cravings in the preceding week was associated with a 3-fold increase in chronic pain and characterized this population as potentially at risk of relapse (45). However, it is worth noting that underdiagnosed or undertreated pain could also result in an imbalance and even an aggravation of opioid abuse disorders.

Secondly, the increase of pain sensitivity (hyperalgesia) induced by long-term opioid use could also form a barrier against opioid prescription in OST patients (46–48). An Australian study has demonstrated that methadone-maintained patients with chronic pain treated with analgesic opioids presented an hyperalgesia when submitted to the cold pressor test (48).

Thirdly, there is wide abuse in terms of diversion associated with analgesic opioids in OST patients, where pain may be reported solely to manipulate prescribers to obtain opioid medication. An American study showed that 57.5% of analgesic opioid misuse occurred in methadone-maintained patients with chronic pain (21). Two recent studies also highlighted that the major source of diverted opioids was physician prescriptions (49, 50).

Finally, some doctors may use methadone and buprenorphine both as substitution therapy and as analgesics. This may partly account for the lack of supplemental opioid analgesic prescription. Moreover it is well known that the concomitant use of high-dose analgesic opioids and OST can lead to increased risk of accidental and fatal overdoses (3, 29). Between 2013 and 2014 in the US, the synthetic opioids overdose-related deaths almost doubled (51). From 1999 to 2015, prescribed opioid overdoses involved more than 183,000 deaths in the U.S (51, 52). In OST patients, overdose mortality accounted for 12.7 overdose deaths per thousand people per year in the U.S.A. (53). The overdose risks are also related to the well acknowledged phenomenon of opioid tolerance that occurs when opioid analgesics are taken over an extended period of time. Patients with opioid tolerance actually require higher opioid dosages to achieve the same analgesic effect, which may increase the risk of accidental overdoses (54–56). Consequently, these potential overdose risks may encourage doctors to avoid adding up opioid drugs or increasing opioid analgesic doses.

Non-or undertreated pain may have negative consequences, including searches for ways to manage pain illegally. Many studies have found that OST patients often felt that their pain was not well managed, and the absence of pain management encouraged them to use illicit opioids for pain relief (25, 27, 35). Many recent studies have reported OST patients using over-the-counter drugs (from 37.7 to 75%) (17, 27), heroin (25%) (27), and non-pharmacological strategies (19% meditation, 12–18% relaxation) (17, 23) to relieve their pain themselves (19–22, 25, 27, 35, 57, 58). Chronic pain should be routinely assessed in OST patients since it constitutes a significant component in the global patient management. The lack of specific recommendations in the assessment and treatment of chronic pain in these patients makes a proper management more complex. It has now been recognized that clinicians require educating about this topic. Despite progress in their training, there are still misconceptions and fears that prevent clinicians from prescribing analgesic opioids in dependent patients that would indeed clearly benefit clinically (44).

### Factors associated with analgesic opioids prescriptions

In multivariate analysis, our study identified several correlations with AOPs in OST patients: female gender, younger age, low-income status, mental health disorder, HCV infection, alcohol use disorder, and MTD treatment. The literature has amply demonstrated that the prevalence of pain is higher in women than in men (12, 59–61). Similarly to our findings, Nosyk et al. found that female gender was associated with significantly higher odds AOP in methadone maintenance treatment (OR = 1.42 [1.31–1.55]) (62). Another recent study found that women were at higher risk of chronic analgesic opioid use following total hip arthroplasty (63). Disparities in healthcare have also been documented (64, 65), with patients of lower incomes more often reporting pain (12, 59, 61, 65) and developing substance abuse disorders (66). Several studies among HCV+ patients reported a high prevalence of pain in this population (34, 67, 68). An American study found that HCV+ patients were frequently diagnosed with pain and substance abuse disorder and were frequently prescribed analgesic opioids (34). More recently, another study showed that the risk of chronic pain in HCV+ patients was doubled (albeit non-significant) (69). Previous studies have identified associations between chronic pain and alcohol dependence (70, 71). According to a recent Australian study, “drinkers” took higher opioid doses, reported more multiple pain conditions, and had higher pain severity than “non drinkers” (70). Some studies reported that patients suffering from depression were more prone to receive chronic opioid therapy, suggesting that mental health disorder might represent a risk factor for opioid misuse (72–76). Grattan et al. reported that there was a 1.8- to 2.4-fold increased risk of opioid medication abuse in patients with depression (77). Moreover, it is well known that mental health disorders are classically associated with chronic pain in substance abuse disorders (19, 23, 24, 78). The coexistence of both a mental health disorder and substance abuse disorder is currently acknowledged to be a co-occurring disorder and refers to the concept of “dual diagnosis” (79, 80). Originally, since the 90s, the term of “triple diagnosis” has been recognized and arose from 1/ a “dual diagnosis”, which is largely acknowledged in the psychiatry field, and refers to a patient with a severe mental illness and a substance use disorder, plus 2/ a “human immunodeficiency virus infection”. Nevertheless, this term of “triple diagnosis” does not have a unique and generally agreed-upon usage since it has also been used in other settings: a few authors introduced the notion of chronic pain as a co-occurring disorder with the earlier “dual diagnosis”. In our study, we are specifically referring to the very same latter meaning. Chronic pain adds a third potential clinical problem in OST patients with mental health disorders and represent the third pathologic dimension of the “triple diagnosis”. These three pathological conditions are so closely intertwined that any positive progression or aggravation of one condition will affect the other two. Overall, this term emphasizes the need to consider treating a patient as a whole and not his component parts separately. The management of this “triple diagnosis” presents a considerable and real challenge for caregivers since its symptoms are often complex and severe.

In our study, 81% of OST patients were prescribed opioids by physicians other than their OST prescriber. These findings are in line with a recent study conducted among methadone-maintained patients, which found that 74% and 67.7% of opioid co-prescriptions with OST originated from non-methadone physicians (62, 81). The reasons behind this disproportion remains unclear; whether opioid prescriptions are obtained for legitimate pain conditions (e.g., dental procedures, chronic pain, trauma, or so on) or for non-medical reasons (e.g., abuse or diversion) cannot be definitively answered and needs further investigation.

## Strengths and limitations

This study represents an original approach in that it is the first to focus on OST patients whose CNCP was treated by analgesic opioids. Furthermore, we used the largest French continuous nationwide claims database (covering 99% of the French population), enabling an exhaustive identification of the OST population in France. Moreover, this is the first European study that provides valuable data about AOPs in OST patients, as France accounts for almost one quarter of all opioid-maintained patients in Europe. In addition, our findings contribute to current knowledge with specific data on prescribed analgesic opioids in CNCP buprenorphine-maintained patients, given that the majority of previous studies were carried out in methadone-maintained patients. Interestingly, unlike others countries, HDB is the most frequent prescribed OST in France, while MTD is the most common treatment elsewhere in Europe and the rest of the world.

Nevertheless, several limitations inherent to claims database are present. First, our data does not include detailed clinical information on for example, severity of addiction, other risks behaviors, or other detailed related to mental disorders that could prevent opioid prescription by doctors. Importantly, characteristics of chronic pain are lacking and particularly no information on pain intensity was available.

The main result of our study shows that OST patients were less frequently prescribed analgesic opioids, compared to a general chronic pain population, a result which may actually simply reflect more severe pain in the latter group. However, this seems unlikely since the literature widely reports higher prevalence of pain in patients with opioid use disorders (31–41 vs. 12–30% in the general population) (15, 16, 24, 27, 82). In this context, we can reasonably assume that the underprescription of analgesic opioids in OST patients may be related to a reluctance of prescribers. In this context of inadequate pain relief, several studies have showed that a significant proportion of OST patients use street-bought or illicit drugs to effectively alleviate their pain.

## Conclusion

AOPs were significantly less frequent and at lower dosages in CNCP OST patients compared to a CNCP control group. We proposed a number of hypotheses that might account for these differences, but we have to remain cautious since confounding bias cannot be totally ruled out. Proper management of patients with a “triple diagnosis” (mental disorders, addiction, and chronic pain), is challenging, and such patients should have access to a multidisciplinary care model thereby avoiding the so-called “wrong door syndrome.” If undiagnosed or untreated, one of these conditions could result in an imbalance and even an aggravation of the other two. The real challenge in OST patients, therefore, is to guide the management of chronic pain through a multidisciplinary approach involving general practitioners, addictologists, psychiatrists, and pain specialists, all working in concert using a syndrome-based approach.

## Ethics statement

No ethic committee approval was necessary since this study used anonymized data from the French national claims database.

## Author contributions

JD wrote the first draft of the manuscript. The data were analyzed by JD and MR. NA, AE, DA, CB and CC provided revision of the intellectual content and final approval of the manuscript.

### Conflict of interest statement

The authors declare that the research was conducted in the absence of any commercial or financial relationships that could be construed as a potential conflict of interest.
